# Impact of Vaccination on the Sense of Security, the Anxiety of COVID-19 and Quality of Life among Polish. A Nationwide Online Survey in Poland

**DOI:** 10.3390/vaccines9121444

**Published:** 2021-12-07

**Authors:** Mateusz Babicki, Wojciech Malchrzak, Anna Hans-Wytrychowska, Agnieszka Mastalerz-Migas

**Affiliations:** Department of Family Medicine, Wroclaw Medical University, 51-141 Wroclaw, Poland; wojciech.malchrzak@gmail.com (W.M.); anna.hans-wytrychowska@umw.edu.pl (A.H.-W.); agnieszka.migas@gmail.com (A.M.-M.)

**Keywords:** COVID-19 vaccination, mental health, GAD-7, quality of life, attitudes towards vaccination

## Abstract

The pandemic state has a destructive effect on the human psyche and induces fear for one’s own health. By reducing the risk of severe COVID-19, vaccination may indirectly improve the mental state. This study aims to assess the effects of vaccination on respondents’ mental well-being, their attitudes towards adherence to government recommendations limiting viral transmission, and to identify factors that may influence the decision to get vaccinated. The survey took the form of the authors’ own, fully voluntary, anonymous, online questionnaire. Standardised psychometric tools were used in the survey: Generalised Anxiety Disorder Assessment (GAD-7) and Manchester Short Assessment of Quality of Life (MANSA). The survey involved 1696 respondents, the vast majority of whom were women, and were aged 18–29. The vaccination status was declared by 1677 respondents (98.9%), 430 (25.4%) of whom were vaccinated with at least one dose of vaccine, while 303 (17.9%) respondents were not only unvaccinated at all, and declared no intention to get vaccinated in the future. Fully vaccinated individuals were found to have lower levels of anxiety, higher MANSA scores and lower subjective anxiety about being infected with COVID-19 than those awaiting vaccination or those with an incomplete vaccination regimen (one dose). Those who are not willing to get vaccinated have the lowest sense of anxiety and fear of being infected and they have the lowest adherence to government recommendations limiting SARS-CoV-2 transmission. Conclusions: COVID-19 vaccination reduces the level of anxiety about being infected and anxiety due to COVID-19 disease in people from the immediate environment. Those who are not willing to get vaccinated have extreme attitudes that negate the pandemic as a whole, including the need for COVID-19 vaccination. Fully vaccinated individuals still adhere to the SARS-CoV-2 prevention policies in place.

## 1. Introduction

The COVID-19 pandemic, which has existed for more than a year, has become one of the biggest public health issues. Numerous cases of SARS-CoV-2 infection have frequently paralysed the healthcare system in many countries worldwide, causing the death of many people every day [1]. According to the latest statistics, more than 180 million cases of SARS-CoV-2 infection have been found and approximately 4 million of them ended in death [2]. However, SARS-CoV-2 infection is not limited to somatic symptoms. It also directly affects the central nervous system, leading to acute psychotic symptoms [3]. SARS-CoV-2 infection also indirectly affects the mental state. The spectre of infection and contraction of the COVID-19 virus, the economic and social situation, as well as social isolation also significantly affect the mental state, which may be manifested by an increased sense of anxiety, including generalised anxiety, and increased depressive symptoms, sleep disorders, and a sense of reduced quality of life [4]. Pre-existing mental health disorder, especially depressive and anxiety disorders, is a significant factor that directly affects mental well-being [5,6,7]. 

Currently, remdesivir is recommended for treating COVID-19; however, it is used only in hospitalised patients [8]. Therefore, the scientific world has focused mostly on the development of effective vaccines, which are currently the only specific way that significantly prevents COVID-19 or at least reduces the risk of severe course of the disease, as well as reduces COVID-19-related hospitalisation and death [9]. After months of testing, the first preparations were conditionally approved for use in late 2020, enabling immediate implementation of vaccination programmes for the population in many countries [10]. Four preparations against COVID-19 are approved for marketing in the European Union Comirnaty (Pfizer/BioNTech, Pfizer, New York, NY, USA) and Spikevax (Moderna, MA, USA) based on mRNA, and Vaxzevria (AstraZeneca, Cambridge, England) and COVID-19 Vaccine Janssen (Johnson & Johnson, New Brunswick, NJ, USA) based on adenoviral vector [11]. These preparations differ in terms of mechanisms of action and, thus, in terms of efficacy against different variants of SARS-CoV-2 [12,13]. The main advantage of vaccines is that they dramatically reduce the risk of severe COVID-19 and COVID-19-related death [14]. However, they do not completely protect against mild or asymptomatic COVID-19 infections. This is a tremendous challenge due to the fact that asymptomatic or slightly symptomatic individuals may represent an important chain of SARS-CoV-2 transmission and pose a risk to unvaccinated individuals [15]. Furthermore, the impact of vaccination on virus transmission is one of the key issues that need further research. 

Previous studies have revealed that COVID-19-related fear and anxiety are associated with greater willingness to vaccinate, but still there is a significant percentage of the population that is unwilling to get vaccinated [16,17,18,19]. Usually, the main reason is anxiety about side effects and lack of confidence in the effectiveness of vaccination [20]. To date, however, the way that vaccination itself alters the mental state has not been investigated. This impact may be significant, as vaccinations, which reduce the risk of death and hospitalization due to COVID-19, may also indirectly reduce the feeling of anxiety and improve the quality of life. However, as previously mentioned, this phenomenon has not been investigated. Nevertheless, it is known that vaccinated individuals do not abandon the recommendations regarding SARS-CoV-2 prevention [21]. Willingness to be vaccinated is also strongly connected to knowledge about vaccine and, secondly, to previous vaccination against influenza [22].

In Poland, vaccination against COVID-19 is free. Initially, the vaccine was available to limited group of citizens, mainly medical professionals. Subsequently, a vaccination program was extended to other groups, firstly the oldest people, and then gradually younger ones. In parallel, some professional groups, such as teachers, or patients with haematological or oncological diseases got quicker access to vaccination. Nowadays, access to the vaccine is common and anyone who want to be vaccinated can do so without problems.

Therefore, this study aims to assess the effects of vaccination on respondents’ mental well-being, their attitudes towards adherence to government recommendations limiting viral transmission, and to identify factors that may influence the decision to get vaccinated. 

Based on previous reports [4,5,6,7], the following research hypotheses were made: (1) fully vaccinated individuals have lower levels of anxiety compared to the unvaccinated or the single-dose vaccinated. (2) Individuals who are not willing to get vaccinated are less likely to adhere to recommendations regarding COVID-19 prevention. (3) Attitudes towards vaccination will depend on a respondent’s level of education, place of residence, and occupation. (4) Being infected with COVID-19, COVID-19 infection of someone close to the respondent, and potential exposure to the infection increases a respondent’s willingness to vaccinate. At the time of study, there were no papers referring directly mental well-being of individuals who received the COVID-19 vaccine. This is the first study that assesses that subject which makes it innovative.

## 2. Materials and Methods

### 2.1. Methodology

The CAWI (Computer-Assisted Web Interview) survey, based on the authors’ own questionnaire distributed through social media, was a fully voluntary, anonymous questionnaire for all individuals aged 18 or older who live in Poland and have access to the internet. The questionnaire in polish was distributed mainly via Facebook. Promotion of the post took place within groups related to COVID-19, vaccination, and COVID-19 vaccinations in Poland. That questionnaire was also spread to various general groups to avoid group selection bias. Data were collected from 20 March 2021 until 30 April 2021, during the peak of the third wave of the COVID-19 pandemic in Poland. Daily incidence rates ranged from 6802 to 35,246 COVID-19 cases, and deaths fluctuated between 428 and 954, reaching the highest values since the beginning of the pandemic in Poland [23]. This was also a period of intense promotion of vaccination against COVID-19 for the population, and the period of questionnaire distribution coincided with the population vaccination rate of approximately 15% (at least one dose), of which approximately 7.8% of vaccinated individuals completed the vaccination regimen [24]. 

Before taking part in the study, the respondents were informed about its methodology, objectives, and estimated duration. Informed consent to participate in the study was then obtained. There was a possibility for the participants to opt out of the study at any stage without disclosing any reason. The study was approved by the Bioethics Committee of the Wroclaw Medical University and was conducted in accordance with the Declaration of Helsinki. 

The authors’ own questionnaire consisted of two parts. The first part concerned questions assessing sociodemographic status including age, sex, place of residence, level of education, marital status, past medical history concerning COVID-19, and COVID-19 vaccination status. Subsequently, the comparison was made between the subjective sense of anxiety about being infected with COVID-19 and anxiety about being infected with other somatic conditions. Questions based on a 10-point Likert scale were used for assessing the level of anxiety about being infected with COVID-19, anxiety about neighbours in quarantine, or neighbours being infected with COVID-19 (1, no anxiety; 10, extreme anxiety). The scale in question was also used for assessing the adherence to current recommendations regarding COVID-19 prevention. The proprietary part of the questionnaire has not been validated and no pilot study has been conducted. Family medicine doctors with the support of a psychiatrist and psychologist participated in the design of the questions.

Another part of the questionnaire consisted of standardised psychometric tools, such as the Generalised Anxiety Disorder Assessment (GAD-7) and Manchester Short Assessment of Quality of Life (MANSA). 

The GAD-7 is a self-report, seven-item psychometric tool designed for assessing symptoms that define anxiety. Each question concerned the frequency of a specific anxiety symptom that was rated on a 4-point Likert scale (0, not at all; 1, some days; 2, most of the time; and 3, almost every day) within the past 14 days. A total score ranging from 0 to 21 was analysed. This tool can also be analysed based on cut-off points, where a score of 5, 10, or 15 points indicates the presence of mild, moderate, or severe anxiety, respectively. A score of at least 10 points indicates a high probability of generalised anxiety disorder [25]. This tool has high internal consistency, obtaining a Cronbach’s Alpha coefficient value of 0.92. 

The Manchester Short Assessment of Quality of Life (MANSA) is a tool for the subjective quality-of-life assessment, along with individual aspects of life. MANSA was based on the Lancashire Quality of Life Profile (LQLP), while maintaining its psychometric parameters. It consists of sixteen questions assessing several aspects of human life. Twelve questions are based on a seven-point Likert scale (from 1 to 7 points). The next four questions are based on yes/no answers. This tool can be analysed as a total score; the more points, the higher the rated quality of life. However, assessments can also be made at the individual question level. The maximum number of possible points is 93 [26,27]. This tool has high internal consistency, obtaining a Cronbach’s Alpha co-efficient value of 0.851. The Polish version of the tool was prepared in the Department and Clinic of Psychiatry at the Wroclaw Medical University in 2000. 

### 2.2. Statistical Analysis

The statistical analysis was conducted using Statistica, version 13.3 (StatSoft, Possmoorweg 122301, Hamburg, Germany). Variables were of qualitative and quantitative nature. A chi-squared test was used for determining the relationships between the compared ordinal variables. Basic descriptive statistics were developed for variables based on interval scales. The normality of distributions for these variables was assessed using three different statistical tests: Kolmogorov-Smirnov test, Lilliefors test and Shapiro–Wilk test. The analysis of statistical significance for variables not meeting the criterion of normality of the distribution of the differences between two means was assessed using the non-parametric Mann–Whitney U test or, for more means, the Kruskal–Wallis test. 

In each case, when evaluating statistical significance, a significance level of *p* < 0.05 was accepted.

## 3. Results

### 3.1. Participants

A detailed profile of the study participants is shown in Table 1. Before the questionnaire was completed, each respondent gave informed consent to participate in the study. A total of 1696 questionnaires were included in the analysis. The vaccination status was declared by 1677 respondents (98.9%), 430 (25.4%) of whom were vaccinated with at least one dose of vaccine, while 303 (17.9%) respondents were not only unvaccinated at all, but they declared no intention to get vaccinated in the future.

### 3.2. Relationship between Vaccination and Attitudes/Emotions

A detailed summary of the COVID-19 vaccination status of both the total GAD-7/MANSA scores and individual questions comprising MANSA is shown in Table 2. In adherence to the Ministry of Health recommendations, anxiety about neighbours in quarantine and anxiety about neighbours being infected with COVID-19 are also included. When it comes to fear and anxiety about the pandemic, there is a clearly distinguishable group of individuals who are not willing to get vaccinated. Their declared level of fear and anxiety related to potential SARS-CoV-2 infection is significantly lower than that of the others, whether they have already been vaccinated or not, as confirmed by the post-hoc analysis. Those who are not willing to get vaccinated are also significantly less likely to adhere to government recommendations limiting virus transmission. Fully vaccinated individuals, as opposed to those not yet vaccinated or those vaccinated with one dose, are most likely to adhere to the current recommendations regarding COVID-19 prevention. Given the entire GAD-7 questionnaire, the differences between the groups are on the borderline of statistical significance. The greatest differences were found in three questions included in this questionnaire; in each of these questions, the fully vaccinated rated their anxiety level as lower than other participants. When it comes to the MANSA questionnaire, fully vaccinated respondents were more satisfied with their life compared to the single-dose vaccinated, the unvaccinated, and those not intending to vaccinate (62.81 vs. 60.22 vs. 59.38 vs. 60.26; *p* = 0.0017). Those who were not willing to get vaccinated had a similar total score to the single-dose vaccinated, while those intending to vaccinate obtained the lowest scores. The post hoc analysis revealed a statistically significant difference between the fully vaccinated and those awaiting vaccination (*p* = 0.0006). In the analysis of individual questions included in the questionnaire, the highest subjective sense of security was declared by the fully vaccinated, whereas the lowest was by the single-dose vaccinated (post-hoc analysis *p* = 0.014). The unvaccinated obtained intermediate scores (*p* = 0.022); there were no statistically significant differences between other groups.

### 3.3. Factors Influencing Current COVID-19 Vaccination

Detailed information on demographic, epidemiological, medical, and social variables in relation to the vaccination status are shown in Table 3. Given sociodemographic factors, residents of rural areas, those with a vocational education or a lower level of education, the divorced, non-healthcare professionals, and those who lost income during the pandemic, are significantly more likely to be unwilling to get vaccinated. Psychiatric treatment, including taking psychiatric medications, is not significantly associated with COVID-19 vaccination status. Moreover, a confirmed, or even potential, contact with the virus (carrying out a COVID-19 diagnostic test, being in quarantine, infections in the immediate environment) was associated with vaccination status. Those without such contact were more likely to report unwillingness to vaccinate against COVID-19. Anxiety about being infected with COVID-19 was significantly related to willingness to vaccinate; those reluctant to get vaccinated declared that they were not afraid of COVID-19 infection or they were more afraid of other diseases, such as cardiovascular diseases. Such individuals are also less likely to seek COVID-19-related information and less likely to track daily statistics related to the epidemic.

## 4. Discussion

The results of this study indicate that being vaccinated affects mental well-being and the level of anxiety about SARS-CoV-2 infection. The fully vaccinated have lower levels of anxiety compared to the single-dose vaccinated or the unvaccinated intending to vaccinate. The exceptions are those who are not willing to get vaccinated against COVID-19; their level of anxiety (subjective and GAD-7 score) is significantly lower compared to other groups. The results of the GAD-7 questionnaire, and questions about subjective anxiety about being infected or anxiety about quarantine or isolation of those from the immediate environment, indicate a significant reduction in anxiety in the fully vaccinated compared to the single-dose vaccinated or the unvaccinated intending to vaccinate. The lowest level of anxiety was represented by those who are not willing to get vaccinated. Those differences may be the result of their denial of the COVID-19 pandemic, including the need for vaccination. At the same time, the fully vaccinated have the highest levels of satisfaction with their life, as well as with their mental health and financial situation, both compared to those who are not willing to get vaccinated and the single-dose vaccinated. These variables overlap with factors that increase the probability of making the decision to vaccinate [28]. 

A plausible explanation for why the single-dose vaccinated have a lower sense of security than the unvaccinated may be that at the time of the study, vaccines were not widely available and those who expressed the greatest desire to get vaccinated took advantage of them first, and studies revealed that anxiety about being infected is one of the main drivers of vaccination [18,19]. Those who are not willing to get vaccinated were not afraid of the threat, so their sense of security was not affected as in other groups of respondents. At the same time, despite the reduction in anxiety, the fully vaccinated are still those who adhere to recommendations regarding COVID-19 prevention the most. It should be emphasised that the study was conducted during the first phase of universal vaccination, when those most aware of the threat posed by the pandemic decided to get vaccinated, and, therefore, they are those who understand the need to adhere to the recommendations limiting the COVID-19 spread even after vaccination. Similarly, those not intending to vaccinate are those who are the least likely to adhere to the recommendations limiting the COVID-19 spread. Adherence to these recommendations despite being fully vaccinated is an expression of the awareness that although the fully vaccinated protect themselves from contracting the coronavirus, or at least from severe COVID-19, they can still transmit the coronavirus to others. This attitude of the fully vaccinated may help protect those who have not yet been vaccinated or cannot get vaccinated for various reasons from the COVID-19 infection. 

Among the patient groups discussed, the unvaccinated not intending to vaccinate require special attention. Opponents of vaccination show a different approach from the rest of society, not only to vaccination but to the pandemic in general. There are certainly several reasons for this type of approach, including the rejection of facts confirmed by the scientific method in favour of conspiracy theories, reliance on half-truths and selected scientific sources that prove a predetermined thesis while ignoring material that contradicts this thesis (cherry-picking), as well as a libertarian, or even anarchist, attitude that rejects any restrictions and orders. At the same time, this group may also include those who do not have sufficiently high confidence in vaccines. In order for a person to have no doubts about vaccination, they must be convinced not only of the vaccine itself, but they must also have confidence in the widely understood “system”, politicians, and public figures, something people have a big problem with these days [29]. Those who question the validity of vaccination are also more likely to be advocates of various therapies that, according to them, cure the patient of COVID-19. For example, they believe in the effect of chloroquine despite the lack of evidence for its efficacy in the case of COVID-19 treatment [29]. According to studies, while opponents of vaccination often believe in other conspiracy theories as well, they do not exhibit higher levels of anxiety [30]. These findings are supported by the results of this study, according to which reductions in anxiety occurred in the vaccinated and in those not willing to vaccinate, whose baseline anxiety was at a low level. Studies conducted at different times reveal a worrying trend; before the vaccine against COVID-19 was launched, many more people declared their willingness to vaccinate than now [31]. 

This is an unfavourable factor because vaccine refusers pose an epidemiological threat to those who cannot be vaccinated due to contraindications and cannot acquire individual immunity to COVID-19 infection. This survey reveals that vaccine opponents are not interested in tracking current pandemic statistics, and they do not have a need to expand their knowledge in this area. This may explain why there is no correlation between the number of infections at a given time and the willingness to vaccinate against COVID-19 [32]. On the other hand, these individuals believe in various conspiracy theories from the non-existence of the SARS-CoV-2 virus through the financial benefits charged by healthcare professionals for sustaining the pandemic, spreading the virus via 5G network, and microchips injected into the body along with the vaccine against COVID-19 for unspecified purposes [33,34,35]. Nowadays, social media are one of the primary sources of information on the internet; news services, popular science networks, and, finally, strictly scientific sources are much less popular. All of this makes it possible for everyone to create content that is not necessarily true, which is then copied and reaches many people in a short period of time; this is how “fake news” is frequently created. Fake news causes infodemia, i.e., the flood of excessive amounts of information from which an average person is not able to select the valuable one and reject the false one. Currently, infodemia is one of the most serious public health problems, as the prevailing misinformation effectively hinders the fight against many problems, including the SARS-CoV-2 epidemic [36]. 

To the authors’ knowledge, there is still a lack of data that assess the effects of COVID-19 vaccination on mental well-being and the level of COVID-19-related anxiety. There are also limited data concerning the impact of non-COVID-19 vaccines, which makes this study innovative and significant. 

This study also addresses the evaluation of sociodemographic variables in terms of vaccination rates. The correlation between the willingness to vaccinate against COVID-19 and some socio-demographic characteristics is also reflected in other studies. The level of education is a significant determinant of willingness to vaccinate; better-educated people get vaccinated more willingly, which is most likely related to their greater knowledge about how vaccines work, as well as their greater concern for the welfare of the general public. Factors that are associated with lower willingness to vaccinate include female sex, young age, and low income [37,38,39,40]. The Polish study found that younger age and female sex were associated with lower willingness to vaccinate [41]. Healthcare professionals are significantly more likely to be vaccinated, which is related not only to their knowledge about vaccines but also their direct contact with COVID-19 patients [38,42]. Nevertheless, approximately 10% of healthcare professionals are not willing to get vaccinated, which, in addition to the standard adverse effects of vaccine refusal, may also discourage those who have not yet made a decision, as healthcare professionals’ recommendations have a significant impact on a patient’s decision about being vaccinated [39]. The attitude towards other recommended vaccines in the past, such as seasonal influenza vaccines, turned out to be an essential fact as well. Individuals who were previously positive about vaccination show now their willingness to vaccinate again, whether against influenza or COVID-19 [38,43]. 

Other factors that do not significantly influence willingness to vaccinate include, inter alia, COVID-19 infection in the past. An explanation could be the belief of such individuals that they naturally acquired immunity against the coronavirus, or it could be an administrative obstacle, as at the time of the study there were regulations mandating that a three-month interval be maintained between a positive test for SARS-CoV-2 infection and vaccine uptake [44]. There were also no significant differences among those with a history of, or currently undergoing, psychiatric treatment. While COVID-19 infection in the past does not affect willingness to vaccinate, a loved one being infected with COVID-19 increases this willingness; this variable has also been found in other studies and is likely related to the direct link between the loved one and the disease that the vaccine may protect against, especially if there is daily close interaction that is a potential cause of virus transmission [45]. 

The authors are aware of the limitations of this review, which is undoubtedly the methodology of data collection. Due to the anonymous form of online data collection, it was not possible to determine the response ratio or to determine the number of surveys started and not completed. As identification was not performed, it was not possible to inform participants of the results of the study or provide psychological support if necessary. Furthermore, the questionnaire has not been validated and there was no pilot study. There was an overrepresentation of women in the study, especially those living in a large city, which may have influenced the results. The study group is not representative of Polish society despite the fact that the questionnaire was distributed to various general groups. That is why further research on a representative group is necessary to be able to interpolate the results to the general public. In addition, questionnaire surveys distributed via the Internet are burdened with the risk of group selection error. In order to reduce this risk, the dissemination of information about the survey took place through many groups on various topics.

In summary, COVID-19 vaccination may affect subjective improvement in quality of life as well as affect mental well-being; in particular it may contribute to the reduction of COVID-19-related anxiety, especially that about being infected with COVID-19. Moreover, the fully vaccinated have the highest subjective sense of security. However, because the COVID-19 virus transmission may continue to persist despite vaccination, it is essential that both the vaccinated and the unvaccinated adhere to recommendations limiting the spread of SARS-CoV-2. Ongoing monitoring of this phenomenon is crucial. We recommend the widest possible promotion of vaccination against COVID-19, especially by increasing the level of public awareness, because it is known that the willingness to vaccinate closely correlates with it. [22] This enables us to achieve all the benefits of vaccination for both the individual and public health.

## 5. Conclusions

COVID-19 vaccination reduces the level of anxiety about being infected and anxiety due to COVID-19 disease in people from the immediate environment. Those not intending to get vaccinated have attitudes that negate the pandemic, including the need for COVID-19 vaccination, and their level of COVID-19-related anxiety is significantly the lowest. Fully vaccinated individuals still adhere to the SARS-CoV-2 prevention policies in place.

## Figures and Tables

**Table 1:** Characteristics of the study group.

Variable		Participants (n = 1696)
Sex	Male	302 (17.8%)
	Female	1394 (82.2%)
Age	18–29	1241 (73.2%)
	30–39	227 (13.4%)
	40–59	211 (12.4%)
	≥60	17 (1.0%)
Place of residence	Rural area	326 (19.2%)
	City of over 250,000 inhabitants	749 (44.2%)
	Town of up to 50,000 inhabitants	268 (15.8%)
	City of 50,000–250,000 inhabitants	353 (20.8%)
Level of education	Incomplete higher	543 (32.0%)
	Higher (university degree)	654 (38.6%)
	Secondary	445 (26.2%)
	Vocational	21 (1.2%)
	Lower secondary	24 (1.4%)
	Primary	9 (0.5%)
Marital status	Married	323 (19.0%)
	Single	834 (49.2%)
	Partnership	475 (28.0%)
	Widowed	14 (0.8%)
	Divorced	50 (2.9%)
Healthcare professional	Yes	245 (14.4%)
	No	1451 (85.6%)
Recent home isolation	No	1359 (80.1%)
	Yes, in the past	298 (17.6%)
	Yes, currently	39 (2.3%)
COVID-19 vaccination	No	944 (55.7%)
	No, I do not want to get vaccinated	303 (17.9%)
	Yes, I am fully vaccinated	283 (16.7%)
	Yes, but I am vaccinated with a single dose	147 (8.7%)
	No answer	19 (1.1%)

**Table 2:** Vaccination status versus feelings and attitudes towards the epidemic.

Variables		COVID-19 Vaccination					
		No	No, I Do Not Want to Get Vaccinated	Yes, I Am Fully Vaccinated	Yes, But I Am Vaccinated with a Single Dose	*p*	Effect Size *
Age, M ± SD [years]		26.48 ± 8.49	28.33 ± 9.28	29.03 ± 10.32	33.31 ± 12.87	<0.001	0.043
Anxiety about contracting COVID-19 disease		5.46	2.83	5.04	5.52	<0.001	0.133
Anxiety about contracting COVID-19 compared to other diseases	No anxiety	141 (39.0%)	159 (43.9%)	39 (10.8%)	23 (6.4%)	<0.0001	0.228
	Yes, to a greater extent	160 (63.0%)	5 (2.0%)	51 (20.1%)	38 (15.0%)		
	Yes, to the same extent	324 (60.0%)	53 (9.8%)	112 (20.7%)	51 (9.4%)		
	Yes, to a lesser extent	319 (61.2%)	86 (16.5%)	81 (15.6%)	35 (6.7%)		
Anxiety if it appears that my neighbour is in quarantine.		3.24	1.97	2.78	3.22	<0.001	0.043
Anxiety if it appears that my neighbour has COVID-19 disease.		3.88	2.24	3.38	3.87	<0.001	0.054
Adherence to the Minister of Health’s recommendations regarding COVID-19 prevention.		7.48	4.64	8.14	7.73	<0.001	0.223
**Total GAD-7 score**		10.14	9.64	9.01	9.63	0.050	0.011
Are you generally satisfied with your life now?		4.20	4.35	4.67	4.47	<0.001	0.021
How satisfied are you with your job (other professional activities, school)?		4.06	4.07	4.35	4.50	0.004	0.012
Are you satisfied with your financial situation?		4.07	3.83	4.59	4.20	<0.001	0.022
Are you satisfied with the number and quality of your friendships?		4.44	4.52	4.79	4.45	0.071	0.009
How satisfied are you with your leisure activities (hobbies)?		3.84	3.74	3.93	4.12	0.214	0.002
Are you satisfied with your housing situation?		4.75	4.63	4.99	4.88	0.106	0.004
Do you have a sufficient sense of security?		5.00	4.95	5.13	4.66	0.022	0.025
Are you satisfied with your relationships with the people you live with?		5.16	5.37	5.26	5.03	0.070	0.003
Are you satisfied with your sex life?		3.99	4.31	4.31	3.88	0.007	0.006
How satisfied are you with your relationship with your family?		4.85	4.93	5.08	4.76	0.087	0.003
Are you satisfied with your physical health?		4.07	4.22	4.19	4.15	0.376	0.001
Are you satisfied with your mental health?		3.73	4.05	4.14	3.97	0.002	0.009
**Total MANSA score**		59.38	60.26	62.81	60.22	0.002	0.008

* Eta-squared/Cramer’s V.

**Table 3:** Vaccination status versus sociodemographic, epidemiological, and medical variables.

Variables		COVID-19 Vaccination						
		No	No, I Do Not Want to Get Vaccinated	Yes, I Am Fully Vaccinated	Yes, But I Am Vaccinated with a Single Dose	*p*	Effect Size *	X^2^
Place of residence	Rural area	189 (58.7%)	68 (21.1%)	34 (10.6%)	31 (9.6%)	0.024	0.059	18.062
	Town of up to 50,000 inhabitants	150 (56.8%)	51 (19.3%)	46 (17.4%)	17 (6.4%)			
	City of 50,000–250,000 inhabitants	197 (56.0%)	65 (18.5%)	56 (15.9%)	34 (9.7%)			
	City of over 250,000 inhabitants	408 (55.2%)	113 (16.1%)	147 (19.9%)	65 (8.8%)			
Level of education	Primary	7 (77.8%)	2 (22.2%)	0 (0.0%)	0 (0.0%)	<0.001	0.123	75.991
	Lower secondary	14 (58.3%)	10 (41.7%)	0 (0.0%)	0 (0.0%)			
	Vocational	13 (63.9%)	8 (38.1%)	0 (0.0%)	0 (0.0%)			
	Secondary	275 (62.2%)	97 (22.0%)	45 (10.2%)	25 (5.7%)			
	Incomplete higher	302 (56.2%)	75 (14.0%)	117 (21.8%)	43 (8.0%)			
	Higher (university degree)	333 (51.7%)	111 (17.2%)	121 (18.8%)	79 (12.3%)			
Marital status	Married	145 (45.5%)	68 (21.3%)	63 (19.8%)	43 (13.5%)	<0.001	0.101	52.072
	Single	510 (62.0%)	124 (15.1%)	130 (15.8%)	58 (7.1%)			
	Partnership	258 (54.7%)	96 (20.3%)	84 (17.8%)	34 (7.2%)			
	Widowed	5 (35.7%)	2 (14.3%)	2 (14.3%)	5 (35.7%)			
	Divorced	26 (52.0%)	13 (26.0%)	4 (8.00%)	7 (14.00%)			
Healthcare professional	Yes	42 (17.7%)	23 (9.5%)	156 (64.5%)	21 (8.7%)	<0.001	0.527	467.16
	No	902 (62.9%)	280 (19.5%)	127 (8.9%)	126 (8.8%)			
Deprivation of earning opportunities due to the pandemic	No	723 (54.7%)	221 (16.7%)	254 (19.2%)	125 (9.45%)	<0.001	0.139	32.833
	Yes	221 (62.4%)	82 (23.2%)	29 (8.2%)	22 (6.2%)			
Prior psychiatric treatment	No	748 (55.7%)	250 (18.6%)	233 (17.3%)	113 (8.4%)	0.335	0.044	3.394
	Yes	196 (58.9%)	53 (15.9%)	50 (15.0%)	34 (10.2%)			
Using psychiatric medications	No	789 (56.4%)	260 (18.6%)	235 (16.8%)	116 (8.3%)	0.326	0.045	3.463
	Yes	155 (56.0%)	43 (15.5%)	48 (17.3%)	31 (11.2%)			
Recent suspicion of COVID-19	No	710 (53.5%)	253 (19.1%)	250 (18.8%)	115 (8.7%)	<0.001	0.126	26.934
	Yes	234 (67.1%)	50 (14.3%)	33 (9.5%)	32 (9.2%)			
Recent quarantine	No	733 (55.0%)	250 (18.7%)	240 (18.0%)	111 (8.3%)	0.018	0.676	15.356
	Yes, in the past	187 (59.9%)	48 (15.4%)	43 (13.8%)	34 (10.9%)			
	Yes, currently	24 (77.4%)	5 (16.1%)	0 (0.0%)	2 (6.5%)			
Recent home isolation	No	730 (54.4%)	251 (18.7%)	247 (18.4%)	113 (8.4%)	0.003	0.778	20.018
	Yes, in the past	185 (62.3%)	45 (15.2%)	35 (11.8%)	32 (10.8%)			
	Yes, currently	29 (74.4%)	7 (18.0%)	1 (2.6%)	2 (5.1%)			
Testing for COVID-19	No	663 (60.7%)	236 (21.6%)	105 (9.6%)	88 (8.1%)	<0.001	0.280	132.196
	Yes	281 (48.0%)	67 (11.5%)	178 (30.4%)	59 (10.1%)			
Diagnosed with COVID-19	No	790 (55.8%)	269 (19.0%)	242 (17.1%)	114 (8.1%)	0.016	0.078	10.267
	Yes	154 (58.8%)	34 (13.0%)	41 (15.7%)	33 (12.6%)			
COVID-19 infection in the immediate environment	No	282 (49.7%)	136 (23.9%)	99 (17.4%)	51 (9.0%)	<0.001	0.117	23.346
	Yes	662 (59.7%)	167 (15.1%)	184 (16.6%)	96 (8.7%)			
Search for additional information on COVID-19	No	507 (55.2%)	215 (23.4%)	131 (14.3%)	65 (7.1%)	<0.001	0.167	47.296
	Yes	437 (57.6%)	88 (11.6%)	152 (20.0%)	82 (10.8%)			
Track daily statistics related to the epidemic	No	518 (53.4%)	234 (24.1%)	146 (15.0%)	73 (7.5%)	<0.001	0.187	58.701
	Yes	426 (60.3%)	69 (9.8%)	137 (19.4%)	74 (10.5%)			

* Fi/Cramer’s V.

## Data Availability

The data presented in this study are available on request from the corresponding author.
